# *Hericium erinaceus* mycelium ameliorate anxiety induced by continuous sleep disturbance in vivo

**DOI:** 10.1186/s12906-021-03463-3

**Published:** 2021-12-05

**Authors:** Tsung-Ju Li, Tung-Yen Lee, Yun Lo, Li-Ya Lee, I-Chen Li, Chin-Chu Chen, Fang-Chia Chang

**Affiliations:** 1 Biotech Research Institute, Grape King Bio, Taoyuan, 32542 Taiwan; 2 Department of Veterinary Medicine, National Taiwan University, Taipei, Taiwan; 3 Department of Food Science, Nutrition, and Nutraceutical Biotechnology, Shih Chien University, Taipei, Taiwan; 4 Institute of Food Science and Technology, National Taiwan University, Taipei, Taiwan; 5 Graduate Institute of Brain and Mind Sciences, College of Medicine, National Taiwan University, Taipei, Taiwan; 6 Graduate Institute of Acupuncture Science, College of Chinese Medicine, China Medical University, Taichung City, Taiwan; 7 Department of Medicine, College of Medicine, China Medical University, Taichung City, Taiwan

**Keywords:** *Hericium erinaceus* mycelium, COVID-19, Sleep, Anxiety, NREM

## Abstract

**Background:**

Sleep disruption is a major public health issue and may increase the risk of mortality by ten-folds if an individual is sleeping less than 6 h per night. Sleep has changed dramatically during to the COVID-19 pandemic because COVID symptoms can lead to psychological distress including anxiety. *Hericium erinaceus* mycelium has been widely investigated in both the in vivo studies and clinical trials for its neuroprotective functions because the mycelium contains hericenones and erinacines, which synthesize the nerve growth factor and brain-derived neurotrophic factor (BDNF). Recent in vivo reports have shown showed that erinacine A-enriched *Hericium erinaceus* mycelium can modulate BDNF/TrkB/PI3K/Akt/GSK-3β pathways to induce an antidepressant-like effect. A large body of evidence indicates that erinacine can pass the blood-brain barrier and suggests its neuroprotective function in both peripheral and central nervous systems. Thus, *Hericium erinaceus* mycelium may be a dual-function supplement for sleep disruption improvement while sustaining anxiolytic effects.

**Method:**

To simulate the condition of sleep disruption, the mice were subjected to the tail suspension test (TST) for 15 min every day during the same period for nine consecutive days. Two different doses (75 and 150 mg/kg) of *Hericium erinaceus* mycelium were administered orally 20 min prior to the TSTs before entering the light period of 12:12 h L:D cycle. All sleep-wake recording was recorded for 24 h using electroencephalogram and electromyogram. The elevated-plus-maze and open-field tests were conducted to record the behavior activities.

**Results:**

Consecutive TSTs prior to the light period could cause significant sleep disturbance and anxiety behavior in the elevated-plus-maze experiments. Results showed that administration with *Hericium erinaceus* mycelium at 150 mg/kg ameliorated the rodent anxiety (*p* < 0.05) and reversed the TST-induced NREM sleep disturbance in the dark period.

**Conclusion:**

This is the first in vivo study suggesting that *Hericium erinaceus* mycelium has a dual potential role for anxiety relief through improving sleep disruptions.

**Supplementary Information:**

The online version contains supplementary material available at 10.1186/s12906-021-03463-3.

## Background

Sleep deprivation is an important public issue in many countries. Statistics have shown that approximately 83.6 million adults in the United States sleep less than 7 h a day [1]. Sleep deprivation often causes a lack of energy restoration and metabolite clearance in the brain, which is crucial in sustaining brain function and behaviors [2]. Poor sleep quality as defined by the National Sleep Foundation [3], can often occur from working pressure, stress, anxiety, and financial burden; it eventually affects the formation of long-term memory [4]. Sleep deprivation is believed to be closely related to anxiety levels [5]. Nevertheless, the recent pandemic issue of COVID-19 causing stress and anxiety at the same time have greatly altered the sleep quality [6].

Sleep is divided into two distinct states: rapid eye movement (REM) sleep and non-rapid eye movement (NREM) sleep [5, 7]. A study showed that changes in NREM sleep often correlate with anxiety levels in mice [8]. On the other hand, REM sleep latency is a feature of the early symptoms in depression [9]. Nerve growth factor (NGF) is a key and important modulator in suppressing wakefulness and contributes to the generation of REM sleep [10]. Nevertheless, once anxiety symptoms occur from sleep deprivation, this kind of vicious sleep disruption will hamper the therapeutic efficacy in anxiety patients. Therefore, proper and safe supplementary nutrients are needed for anxiety relief while improving sleep patterns in anxious patients [11].

*Hericium erinaceus* (*H. erinaceus*) has long been used as a medicinal-culinary mushroom [12], which showed various health benefits including anti-aging [13], antioxidative [14], anticancer [15], and neuroprotection [16]. Studies found that the *H. erinaceus* cultured extract contains about more than 15 compounds of the active cyathin diterpenoid [17] with potential neuroprotective effects in the treatments of Alzheimer’s and Parkinson’s diseases [18]. Incubation with *H. erinaceus* erinacine compounds increases NGF gene expression in the human astrocytoma cell line 1321 N1 [19]. Erinacine A-enriched *H. erinaceus* mycelium can induce an antidepressant-like effect by modulating the BDNF/TrkB/PI3K/Akt/GSK-3β pathway in vivo [20]. An in vivo biodistribution study found that erinacine from the *H. erinaceus* mycelium can be found in the brain tissue indicating its capability to pass the blood-brain barrier and exert a neuroprotective function [21]. A double-blind placebo-controlled study further showed that consumption of three *H. erinaceus* mycelium capsules per day (containing 5 mg/g of erinacine A) can prevent early Alzheimer’s disease versus a placebo group [22]. All these studies suggest that *H. erinaceus* mycelium is safe and effective at improving life quality for patients with neurodegeneration.

Despite the accumulated evidence that *H. erinaceus* has in terms of anti-depression and memory improvement [23], there is no report to date that has investigated the potential of *H. erinaceus* mycelium for sleep deprivation with early anxiety symptoms. The aim of this study is to investigate the alterations of behavior activities and monoamines in a sleep disrupted mouse model treated with nutrient *H. erinaceus* mycelium. We then evaluated its potential as an anxiety relief supplement that can improve sleep.

## Methods

### Preparation of the *H. erinaceus* mycelium

The *H. erinaceus* mycelium was prepared according to previous studies [24]. In brief, the *H. erinaceus* strain was purchased from the Bioresources Collection and Research Center in the Food Industry Research and Development Institute (BCRC 35669; Hsinchu, Taiwan). Initial culture was grown in a 2-L flask using a shaker at ~ 120 rev/min at 25 °C for 5 days containing growth medium at pH 4.5 (0.05% MgSO_4_, 0.25% peptone, 0.5% soybean powder, 4.5% glucose, and 0.25% yeast extract). The seeding medium is then transferred to 500-L fermenters for 5 days and scaled up to 20-ton fermenters for another 12 consecutive days. This large-scale process is then harvested and lyophilized to remove excessive water. The final product is ground into powder form and stored in desiccators at room temperature for in vivo experiments. To evaluate the main chemical composition of *Hericium erinaceus* Mycelium, high-performance liquid chromatography (HPLC) was used to evaluate the active peaks’ contents. Major composition such as erinacine A and erinacine C were analyzed according to previous studies with minor modifications [24, 25].

### Feeding condition

All animal experiments were carried out in compliance with the ARRIVE guidelines and were approved by the National Taiwan University Institutional Animal Care and Use Committee (approval number NTU-107-EL-00182). The C57BL/6 mice used in this study were obtained from BioLASCO (TaiwanCo., Ltd.). Oral gavage was employed for the drug delivery. Two different doses of *H. erinaceus* mycelium (75 and 150 mg/kg) were administered. All administrations of substances were administered 20 min prior to the tail suspension test (TST) before entering the light period of 12:12 h L:D cycle.

### Tail suspension test (TST)

The TST was executed from the beginning of the light period and lasted for 15 min every day and the procedure was performed for nine consecutive days during the same time point. Noise and light were avoided while the tail suspension test experiment was conducted. The environment background was set to the white color during the behavioral task to enhance the contrast of the mice. During the TSTs, the struggle movement was determined offline by EthoVision XT software (Noldus Information Technology, Inc., USA). The TST is one of the most widely used models to assess depression in rodents. Previous studies have shown that the TSTs is an acute stressor that causes sleep disruptions in mice [26].

### Elevated plus maze (EPM) behavior test

The EPM was conducted with a similar method as described previously [20]. In brief, the apparatus was placed at a height 50 cm above the floor with two open arms (50 cm × 9 cm) and two enclosed arms (50 cm × 9 cm × 5 cm). The common central area is 9 × 9 cm. Time spent in both open and closed arms was recorded by the video camera. All paths were wiped with 70% ethanol between each individual test to avoid disturbance due to the scent of the previously tested animal.

### Open field test (OF)

The open field test (OF) was conducted in an acrylic box (60 cm × 60 cm × 20 cm) with a floor divided equally with 96 squares. A digital video camera was fixed vertically above the cage to record the mouse activities. For every mouse analysis, the cage was cleaned with water to remove scent of the previously tested animal to prevent any bias.

### Sleep recording and analysis

Two wire electroencephalogram (EEG) electrodes were surgically implanted on the right frontal lobe and the left occipital lobe. The occipital electrode served as the reference. Two electromyogram (EMG) electrodes were inserted into the neck muscle. The sleep-wake activity was recorded for 24 h after the manipulations. Based on the previous study [27], the animal’s vigilance states were classified to NREM sleep, REM sleep, or wakefulness. Briefly, NREM sleep was characterized by large-amplitude EEG slow waves, high power density values in the delta frequency band (0.5–4.0 Hz), and a relaxed muscle tone from EMGs. During REM sleep, the amplitude of the EEG was reduced, and the predominant EEG power density occurs within the theta frequency (6.0–9.0 Hz). The EMGs exhibit muscle atonia with low EMG amplitudes.

The animals are generally active during wakefulness. There are protracted body movements with robust EMG amplitudes. The amplitude of EEGs is like that observed during REM sleep, but power density values in the delta frequency band are generally greater than those in theta frequency band. The percentage of NREM sleep and REM sleep, slow wave activity during the NREM sleep, and sleep architectures were determined. Animals were housed in individual recording cages in an isolated animal room with the temperature maintained at 23 ± 1 °C and a light:dark (L:D) cycle of 12:12-h (20 W × 6 tubes illumination). Food and water were available ad libitum.

### Plasma dopamine analysis

After the behavioral tests, all mice were sacrificed by perfusion with carbon dioxide (CO_2_) and decapitation. The blood was quickly obtained from the mouse’s heart and stored at − 80 °C until the dopamine levels were measured. The enzyme-linked immunosorbent assay (ELISA) kit for dopamine was obtained from Wuhan Fine Biotech Co., and the detailed procedure followed the manufacturer’s instructions. The absorbance was determined by ELISA microplate (Multiskan EX, Thermo Electron Corp., Waltham, MA) with an O.D. absorbance at 450 nm. The sensitivity is < 0.938 ng/ml, and the assay range is between 1.56 and 100 ng/ml. The intra-assay CV is < 8% and the inter-assay CV is < 10% (manufacturer’s specifications).

### Western blot analysis

The mouse brain tissue was homogenized with RIPA buffer containing phosphatase and protease inhibitors. The total protein content was quantified using Pierce™ BCA Protein Assay Kit (23,227, Thermo, USA). Protein lysates were separated with 10% SDS–PAGE gel by electrophoresis and transferred to polyvinylidene difluoride (PVDF) membranes (ISEQ00010, Millipore, USA) using electroblotting transfer tank (Bio-Rad). The PVDF membrane was blocked with 5% non-fat milk powder in tris-buffered saline with Tween 20 (TBST). Primary antibodies, including BDNF (ab108319; Abcam) and GAPDH (SC-32233; Santa Cruz Biotechnology), were incubated with the membranes diluted at 4 °C overnight. Secondary antibody (65–6120, 62–6520; Invitrogen) was applied and the bound-protein bands were visualized using enhanced chemiluminescence (K-12045-D50, Advansta, USA) and quantified using detection system (BIO-RAD ChemiDoc XRS+, USA). The target protein relative intensity was normalized against GADPH.

### Statistical analysis

The percentages of time spent in NREM sleep, REM sleep, the immobility during the TSTs, the open arm during EPM, and the inner area during the OF were represented as the mean ± standard error of mean (SEM) with indicated sample sizes. This study used two-way analysis of variance (ANOVA) to measure the difference between each group with a *post-hoc* comparison. A level of *p* < 0.05 was considered to indicate a statistically significant difference.

## Results

### The effects of the consecutive TSTs on sleep-wake activity

**Fig. 1:**
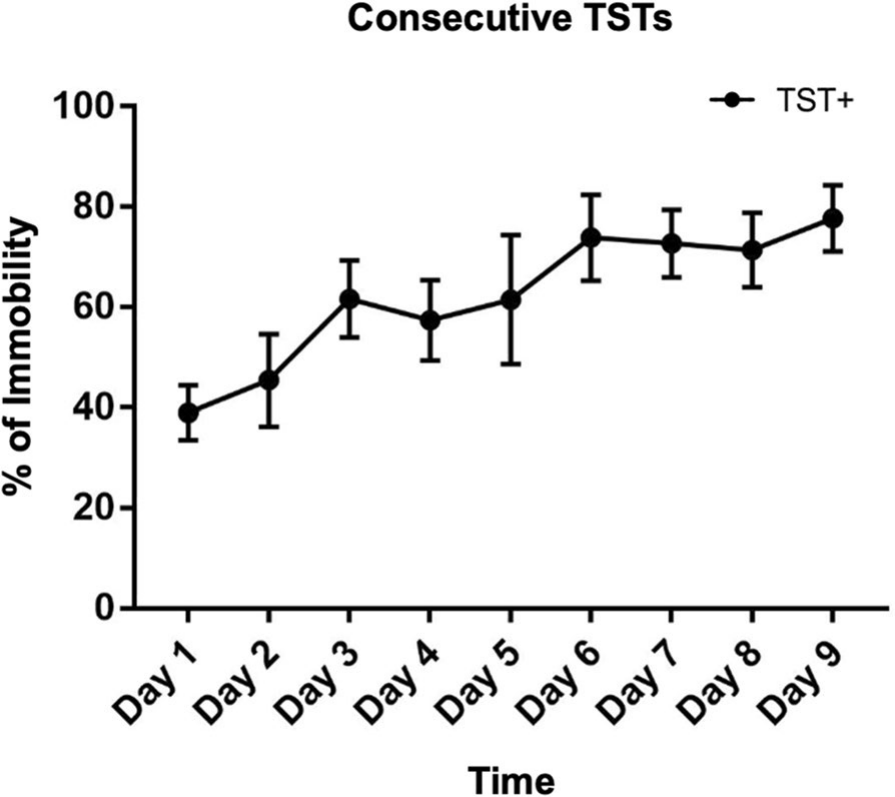
The mice cumulative immobility analysis after the consecutive 9-day TSTs (*n* = 6)

The consecutive 9-day TSTs resulted in an increase in animal immobilization by 40% (Fig. 1) demonstrating that continuous stress can lead to a more emotional helplessness behavior in depression.

**Fig. 2:**
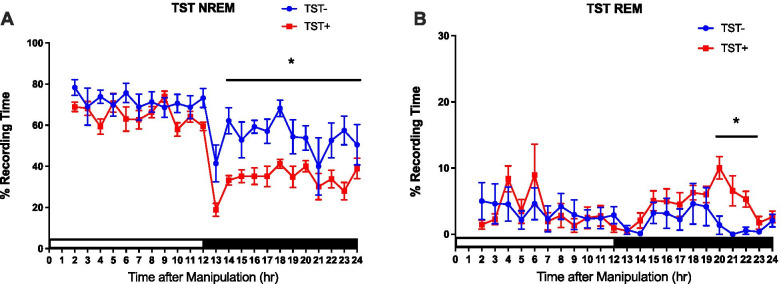
**A** The blue circles represent NREM sleep acquired from the control group without the TSTs (*n* = 6), and the red squares represent the data obtained from the group with the TSTs (*n* = 6). The x-axis depicts the time after the TSTs, and the y-axis represents the percentages of NREM sleep. **B** The blue circles represent REM sleep acquired from the control group without the TSTs (*n* = 6), and the red squares represent the data from the group with the TSTs (*n* = 6). The x-axis depicts the time after the TSTs, and the y-axis represents the percentages of REM sleep. The white and black bars demonstrate the 12 h light period and 12 h dark period, respectively. All data with a * sign means *p* < 0.05

We manipulated the TSTs prior to the light period to further evaluate whether the consecutive TSTs affects sleep-wake activity. The results showed that the TSTs conducted before the light period significantly decreased NREM sleep in the subsequent dark period (hours 13–24) (Fig. 2A), and REM sleep was significantly increased in the late stage of the dark period (hours 20–22) (Fig. 2B).

The analytical results indicate that consecutive TSTs at the beginning of the light period could induce sleep disturbance. The TSTs during the light period could cause a decline in NREM sleep and a rise in the REM sleep in the subsequent dark period.

### The effects of *H. erinaceus* mycelium on sleep-wake activity

**Fig. 3:**
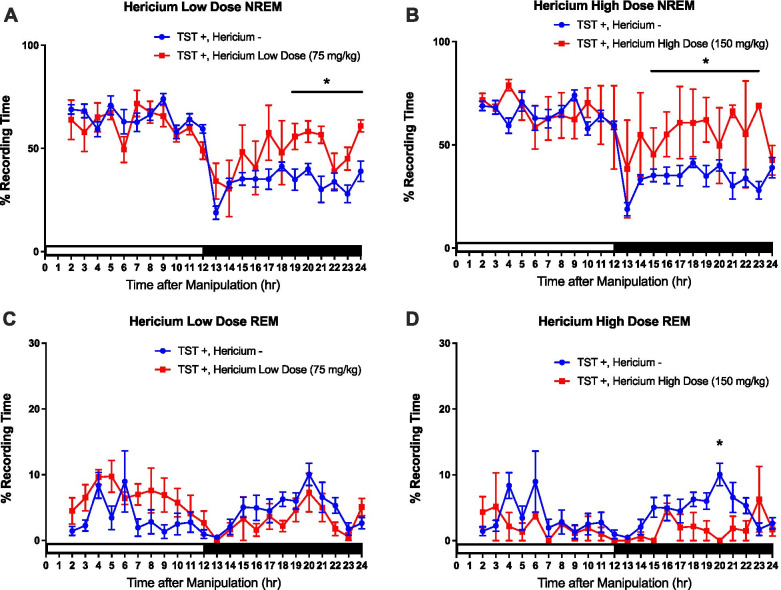
The blue circles represent the sleep data acquired from the TSTs group (*n* = 6), and the red squares represent the data from the group with the low dose of *H. erinaceus* mycelium administration before TSTs (**A**; *n* = 4), and the high dose of *H. erinaceus* mycelium administration (**B**, *n* = 2). The x-axis depicts the time after the TSTs, and the y-axis represents the percentages of NREM sleep. In REM sleep, panel **C** represents the data acquired from a low dose of *H. erinaceus* mycelium administration before the TSTs (*n* = 4), and **D** represents the data acquired from with a high dose of *H. erinaceus* mycelium administration before the TSTs (*n* = 2). The x-axis depicts the time after the TSTs, and the y-axis represents the percentages of REM sleep. The white and black bars represent the 12 h light period and 12 h dark period, respectively. All data with a * sign imply *p* < 0.05

The *H. erinaceus* mycelium was given prior to the light period of a 12:12 h L:D cycle in the TSTs-treated mice. The results showed that both 75 mg/kg and 150 mg/kg of *H. erinaceus* mycelium could increase the percentage of time spent in NREM sleep during the dark period. The effect of *H. erinaceus* mycelium on NREM increases showed up at the late stage of the dark period (hours 19–24) in the low dose (75 mg/kg) group (Fig. 3A). The rise of NREM sleep appeared nearly during the 12 h dark period (hours 15–24) in the high-dose (150 mg/ kg) group (Fig. 3B). Nevertheless, administration of 75 mg/kg *H. erinaceus* mycelium exhibited no effect on TSTs-induced increase of REM sleep; meanwhile there was a REM sleep decline at hour 20 in the group treated with 150 mg/kg of *H. erinaceus* mycelium versus the TSTs group (Figs. 3C and D).

These results suggest that the high dose (150 mg/kg) of *H. erinaceus* mycelium could block the TSTs-induced decreases of NREM sleep; it also increased REM sleep in the dark period. However, the low dose (75 mg/kg) of *H. erinaceus* mycelium could only block the TSTs-induced decreases of NREM sleep in the dark period.

### The effects of *H. erinaceus* mycelium on EPM activity

**Fig. 4:**
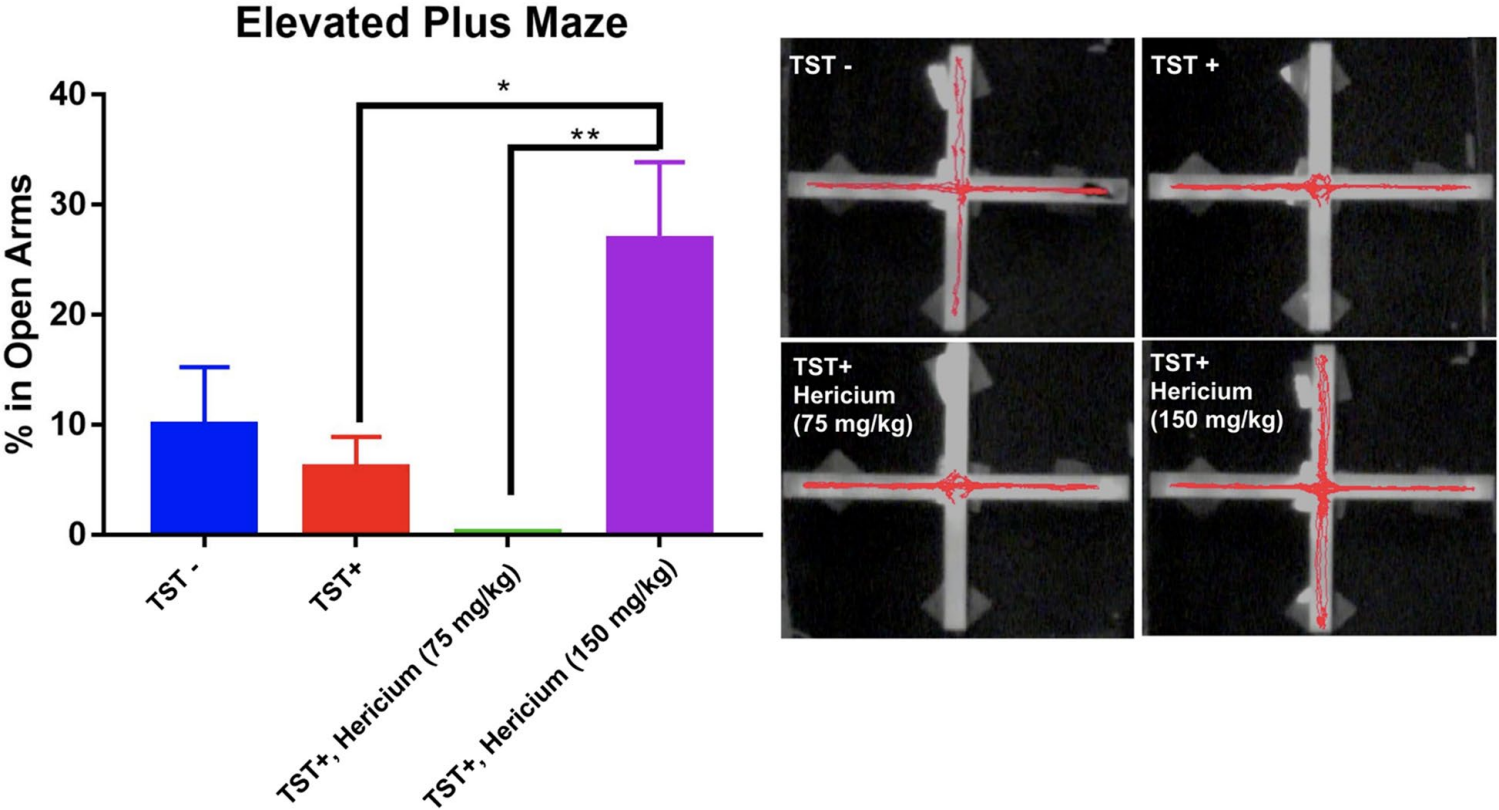
The effects of different doses of *H. erinaceus* mycelium in EPM tests after the TSTs. *: *p* < 0.05

In the EPM experiments, *H. erinaceus* mycelium was administered prior to the light period of the 12:12 h L:D cycle. The results showed that 150 mg/kg of *H. erinaceus* mycelium could increase the proportion of time spent in the open arms compared with that of the TST+ group (Fig. 4). However, lower doses of *H. erinaceus* mycelium (75 mg/kg) showed no anxiolytic effects.

### The effects of *H. erinaceus* mycelium in OF test

**Fig. 5:**
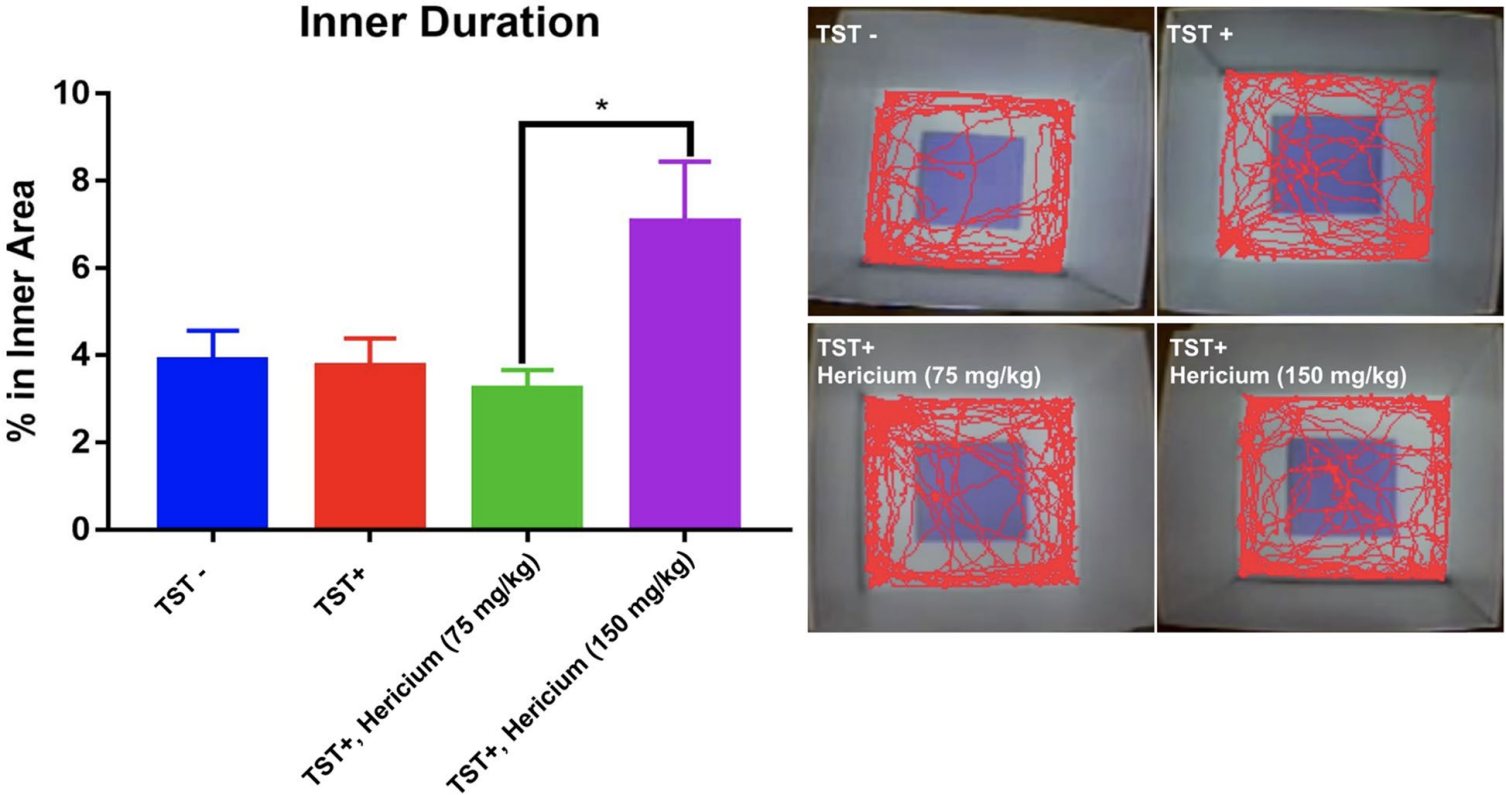
The effects of different doses of *H. erinaceus* mycelium in OF tests after the TSTs. *: *p* < 0.05

In the OF test experiments, *H. erinaceus* mycelium was administered prior to the light period of the 12:12 h L:D cycle. The results showed that 150 mg/kg of *H. erinaceus* mycelium could increase the proportion of time spent in the open arms compared with that of the TST+ group (Fig. 5). However, lower doses of *H. erinaceus* mycelium (75 mg/kg) also showed no anxiolytic effects.

### The effects of *H. erinaceus* mycelium in plasma dopamine activity and brain tissue BDNF

**Fig. 6:**
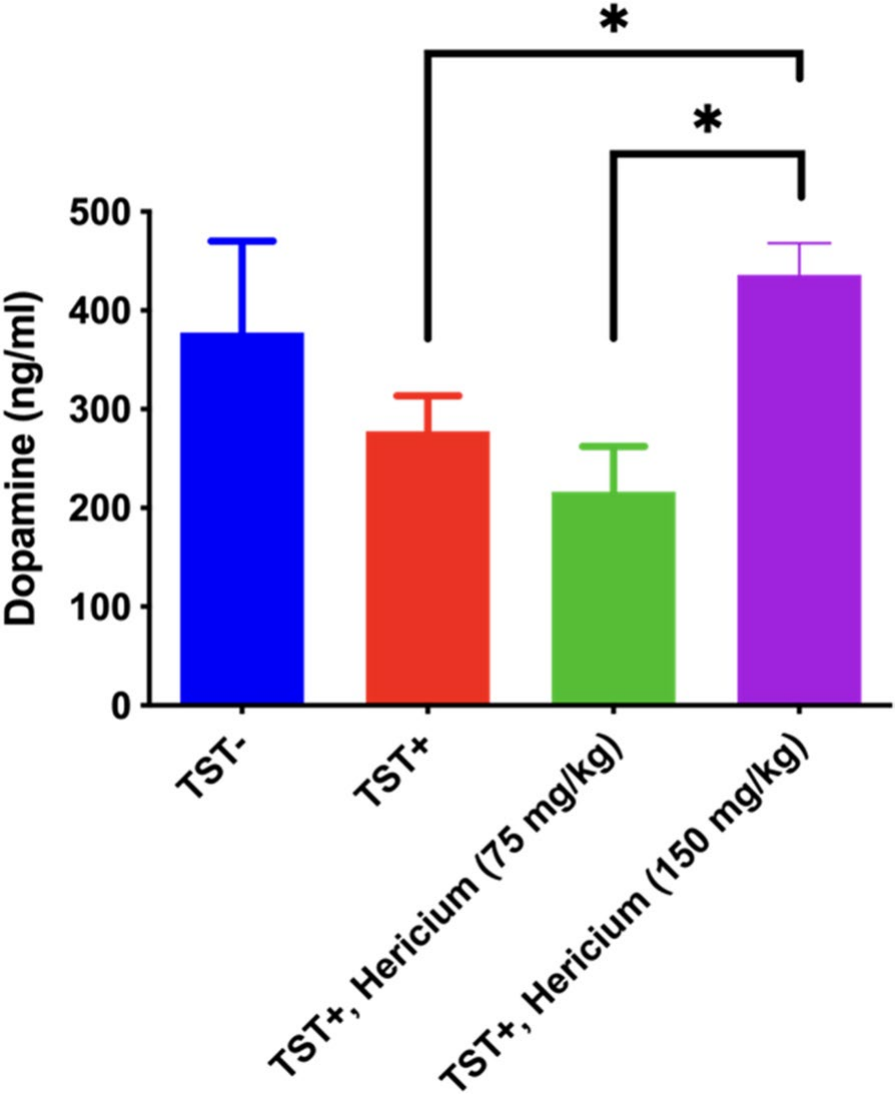
The graph represents the effects of two doses of *H. erinaceus* mycelium in plasma dopamine concentrations. *: *p* < 0.05

In the plasma neurotransmitter analysis, *H. erinaceus* mycelium was administered prior to the light period of the 12:12 h L:D cycle. When TSTs were conducted, the plasma dopamine levels decreased significantly by 26.5%. Low dose of *H. erinaceus* mycelium has no effect in the recovery of the dopamine levels. However, the high dose of *H. erinaceus* mycelium significantly recovered the plasma dopamine concentrations back to normal levels (435.882 ± 32.098 ng/mL, Fig. 6). The mouse brain BDNF expression was also evaluated by western blot analysis. TSTs group showed a significant reduction of BDNF expression after continuous stress. At high dose of *H. erinaceus* mycelium treatment, an increase in BDNF expression can be observed when compared with the control group (Supplementary Fig. S1).

### HPLC analysis of the components from *H. erinaceus* mycelium

**Fig. 7:**
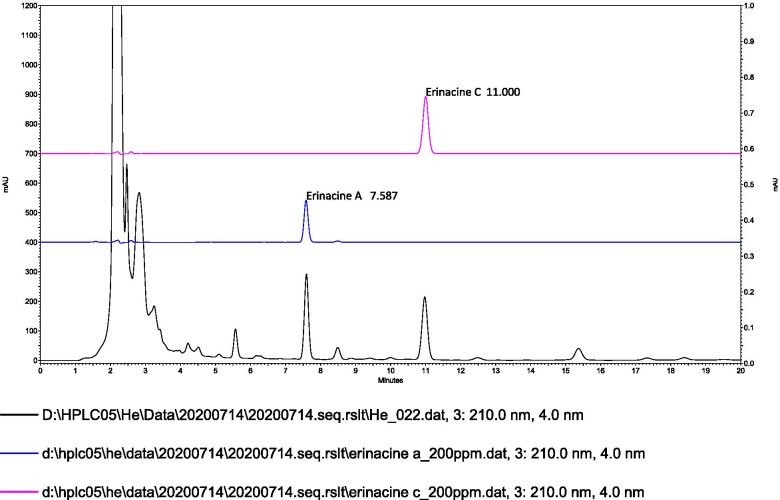
HPLC chromatograms for the standard of erinacine A (blue line), C (purple line), and the *H. erinaceus* mycelium extract (black line)

The identification of the erinacine component was extracted using 85% ethanol from the *H. erinaceus* mycelium (Fig. 7). The erinacine A and erinacine C was confirmed by comparing the characteristics of the spectrum of the sample peak with those of the standard peak (blue line, erinacine A; purple line, erinacine C) with a yield of 7.20 mg/g and 3.35 mg/g, respectively.

## Discussion

Insufficient sleep has become a public health issue according to the US Centers for Disease Control and Prevention (CDC) [28]. Individuals who sleep less than 6 h have a ten-fold increased mortality [29]. Moreover, these issues might have changed dramatically due to the recent COVID-19 pandemic. Researchers have found that COVID-19 is closely associated with individual psychological distress [30]. Of the 1250 self-isolated healthcare professionals during the outbreak, 44.6% of them reported reducing sleep quality because of high anxiety levels [31]. Thus, new tools are needed to reduce anxiety and promote sleep especially in those affected by COVID-19.

Several studies have shown that *H. erinaceus* mycelium can stimulate the synthesis of NGF for neuroprotection and prevents neurodegenerative diseases [32]. Depression is a common neuropsychiatric conditions and abnormal behaviors can be improved by the *H. erinaceus* mycelium in animals [33]. Sleep disturbance is a complicated mechanism, and the TST is a useful method to evaluate sleep deprivation [34]. By using the TST-induced depression model, we showed that the consecutive 9-day TSTs stress performed at the beginning of the light period could generate a significant sleep disturbance while concurrently causing helplessness behavior. Based on the idea that sufficient NREM sleep is essential for brain development and functions [35], we evaluated whether *H. erinaceus* mycelium treatment can ameliorate sleep disturbance in the TSTs-induced depressive animals. Versus TST-induced models without any medication treatment, our results showed that a high dose of *H. erinaceus* mycelium blocked TSTs-induced increases of NREM sleep.

The REM analysis literature has shown that stress-induced sleep deprivation can cause a REM sleep rebound in the dark period [36]. We also observed this REM sleep rebound after consecutive TSTs. We also found that this TSTs-induced REM sleep rebound was reversed back to normal when *H. erinaceus* mycelium was treated. Administration of *H. erinaceus* mycelium at 150 mg/kg alleviates the stress-induced sleep disruptions and supports healthy psychological behavior in animals.

The EPM and OF tests are comprehensive methods to analyze anxiety in mice [37]. In the previous EPM-related studies, *H. erinaceus* mycelium has shown its effectiveness in reducing anxiety [38]. However, no study has yet discussed whether *H. erinaceus* mycelium still maintains its effectiveness on the consecutive 9-day TSTs-induced anxiety and sleep disruptions. In this study, we discovered that animals with consecutive sleep disruptions and treated with TSTs spend more time in the center area and the closed arms in the EPM test. This phenomenon is reasonable because the EPM test itself stimulates the hypothalamic-pituitary-adrenal axis and sympathetic nervous system [39]. Therefore, continuous TSTs stress may cause animals’ behavior to become more hyperresponsive because of the higher corticosterone levels in blood [40].

Here, we demonstrated that higher consumption of *H. erinaceus* mycelium at 150 mg/kg could significantly ameliorate anxiety levels; 75 mg/kg does not offer anxiolytic activity. To demonstrate more evidence that *H. erinaceus* mycelium can maintain its anxiolytic effect under continuous sleep disruption, the OF test is another common platform to analyze the animal’s overall locomotor activity and anxiety-related behaviors [41]. Continuous TSTs do not affect the time spent in the inner area, which may be due to the habituation of repeated handling with a subsequent decrease in height-induced anxiety [42]. Our current study demonstrated that 150 mg/kg *H. erinaceus* mycelium can increase exploration suggesting that mice had lower anxiety than the control [43].

Dopamine is a well-known sleep-wake regulator and is closely linked with the circadian rhythm [44]. Our study found that continuous stress exposure and subsequent sleep disruptions can cause a decrease in dopamine levels. This is different from other studies reporting that chronic stressors cause dopaminergic blunting [45]. On the other hand, we found no difference in the levels of GABA and serotonin (data not shown). Based on these comprehensive behavioral analyses from the EPM and OFT tests, we suggest that the continuous sleep disruption induced by early anxiety from the TSTs can be ameliorated by *H. erinaceus* mycelium through the restoration of the dopamine levels. Our result also elucidated that a high dose of *H. erinaceus* mycelium involves increasing BDNF expression within the brain is an important biomarker for sleep behavioral changes. For the first time, this study also showed that erinacine A and erinacine C in the *H. erinaceus* mycelium can be major compounds and active ingredients. A recent clinical experiment suggested that 8 weeks of oral supplementation of *H. erinaceus* mycelium can improve mood and sleep disorders as determined by the circulating pro-BDNF and BDNF as biomarkers [46]. Future studies should address the interaction between these compounds together with BDNF neurotransmitters to better understand the dual roles of *H. erinaceus* mycelium in both sleep and anxiety. It’s also important to investigate the potential factors, such as other neuroinflammatory pathway that contribute to the efficacy of *H. erinaceus* mycelium.

## Conclusion

Our results indicated that the high dose (150 mg/kg) of *H. erinaceus* mycelium with erinacine A 7.20 mg/g and erinacine C 3.35 mg/g contents reversed the TST-induced sleep disruptions. *H. erinaceus* mycelium also showed its dual potential roles in anxiety relief and sleep improvement. Future clinical trials should address these dual effects of *H. erinaceus* mycelium through a randomized placebo-controlled trial.

## Supplementary Information

**Additional file 1: Figure S1.** Unprocessed western blots images of brain tissue were provided. Each lane represents one mouse brain lysate with treatment as labeled above. The blot was cut prior to hybridization with antibodies for BDNF protein (bottom lane for mature form) and GADPH protein due to close protein band size. The blot intensities were quantified by BDNF to GAPDH expression from the same lysate lane (BDNF lane 1/ GADPH lane 1), for each treatment group total *n* = 3. *: *p* < 0.05.

## Data Availability

The data and raw materials presented in this study are available from the corresponding author upon request.

## Abbreviations

BDNF: Brain-derived neurotrophic factor
EEG: Electroencephalogram
EMG: Electromyogram
EPM: Elevated plus maze
*H. erinaceus*: *Hericium erinaceus* mycelium
HPLC: High-performance liquid chromatography
NGF: Nerve growth factor
OF: Open field
REM: Rapid eye movement
NREM: Non-rapid eye movement
TST: Tail suspension test
