# Supplementary material for: Hericium erinaceus mycelium ameliorate anxiety induced by continuous sleep disturbance in vivo
Source: BMC Complement Med Ther. 2021 Dec 5;21:295. doi: 10.1186/s12906-021-03463-3 (PMC8643634; doi:10.1186/s12906-021-03463-3)
Supplement: Supplementary file 1 — Additional file 1: Figure S1. Unprocessed western blots images of brain tissue were provided. Each lane represents one mouse brain lysate with treatment as labeled above. The blot was cut prior to hybridization with antibodies for BDNF protein (bottom lane for mature form) and GADPH protein due to close protein band size. The blot intensities were quantified by BDNF to GAPDH expression from the same lysate lane (BDNF lane 1/ GADPH lane 1), for each treatment group total n = 3. *: p < 0.05. [file 12906_2021_3463_MOESM1_ESM.docx]

Supporting Information

***Hericium erinaceus* Mycelium Ameliorate Anxiety Induced by Continuous Sleep Disturbance *in vivo***

**Tsung-Ju Li^1^, Tung-Yen Lee^2^, Yun Lo^2^, Li-Ya Lee^1^, I-Chen Li^1^, Chin-Chu Chen^1,3,4^*, Fang-Chia Chang^2,5,6,7^***

^1^ Biotech Research Institute, Grape King Bio, Taoyuan 32542, Taiwan.

^2^ Department of Veterinary Medicine, National Taiwan University, Taipei, Taiwan.

^3^ Department of Food Science, Nutrition, and Nutraceutical Biotechnology, Shih Chien University, Taipei, Taiwan.

^4^ Institute of Food Science and Technology, National Taiwan University, Taiwan.

^5^ Graduate Institute of Brain and Mind Sciences, College of Medicine, National Taiwan University, Taipei, Taiwan.

^6^ Graduate Institute of Acupuncture Science, College of Chinese Medicine, China Medical University, Taichung City, Taiwan.

^7^ Department of Medicine, College of Medicine, China Medical University, Taichung City, Taiwan.

*Correspondence: gkbioeng@grapeking.com.tw (C.-C.C.); fchang@ntu.edu.tw (F.-C. C.)

Contact: Prof. Fang-Chia Chang
Email: fchang@ntu.edu.tw
Address: Department of Veterinary Medicine, National Taiwan University, Taipei, Taiwan (R.O.C.)

^
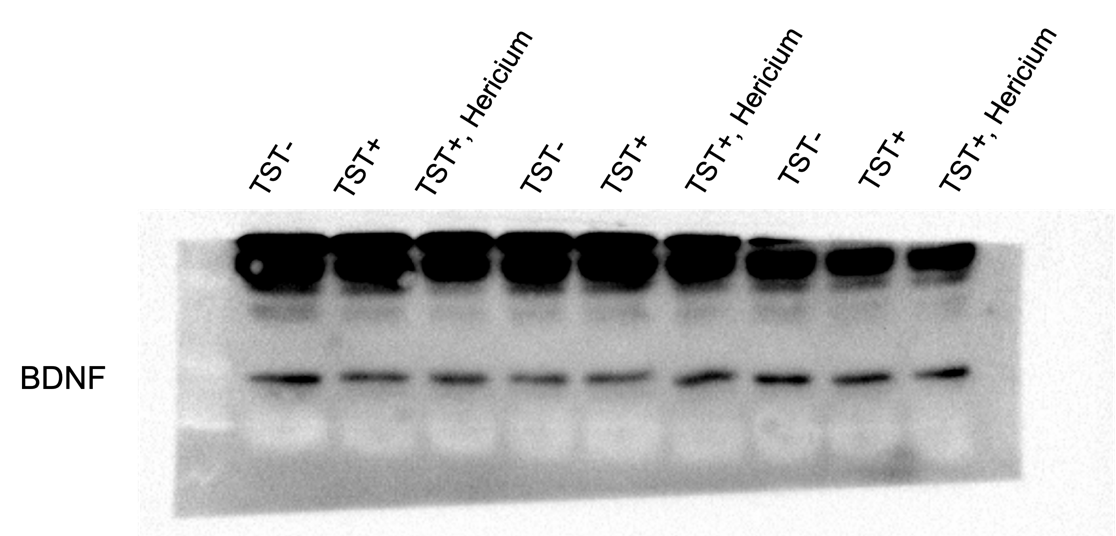
^

^
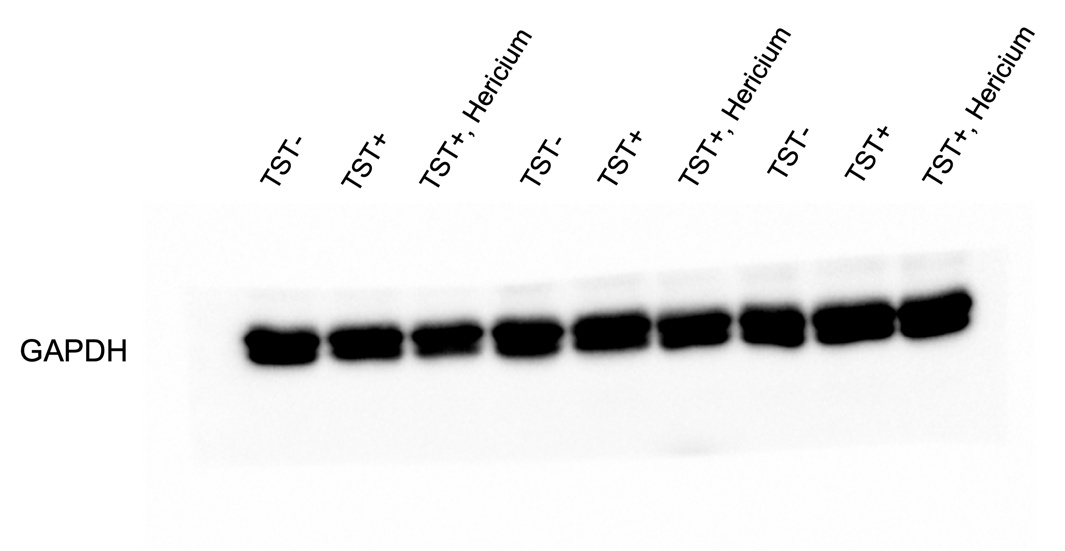
^

^
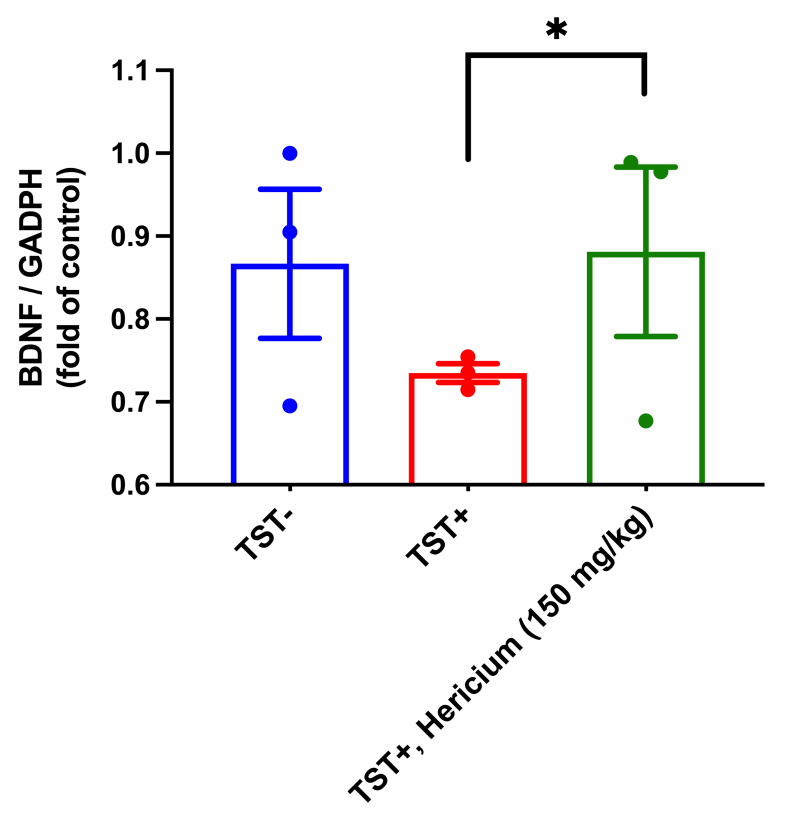
^

**Figure S1**. Unprocessed western blots images of brain tissue were provided. Each lane represents one mouse brain lysate with treatment as labeled above. The blot was cut prior to hybridization with antibodies for BDNF protein (bottom lane for mature form) and GADPH protein due to close protein band size. The blot intensities were quantified by BDNF to GAPDH expression from the same lysate lane (BDNF lane 1/ GADPH lane 1), for each treatment group total n = 3. *: p<0.05
